# A new model for sensitive detection of zoonotic prions by PrP transgenic *Drosophila*

**DOI:** 10.1016/j.jbc.2021.100878

**Published:** 2021-07-13

**Authors:** Alana M. Thackray, Olivier Andréoletti, John Spiropoulos, Raymond Bujdoso

**Affiliations:** 1 Department of Veterinary Medicine, University of Cambridge, Cambridge, UK; 2 UMR INRA ENVT 1225 -Hôtes-Agents Pathogènes, Ecole Nationale Vétérinaire de Toulouse, Toulouse, France; 3 Pathology Department, Animal and Plant Health Agency (APHA), Weybridge, Addlestone, Surrey, UK

**Keywords:** bioassay, classical and atypical BSE, *Drosophila*, infectious disease, mouse, neurodegenerative disease, prion, protein misfolding, PrP^Sc^, BSE, bovine spongiform encephalopathy, CJD, Creutzfeldt–Jakob disease, H-type, heavy type, L-type, light type, PI, performance index, PK, proteinase K, PMCA, protein misfolding cyclic amplification, PrP, prion protein, vCJD, variant CJD

## Abstract

Prions are transmissible protein pathogens most reliably detected by a bioassay in a suitable host, typically mice. However, the mouse bioassay is slow and cumbersome, and relatively insensitive to low titers of prion infectivity. Prions can be detected biochemically *in vitro* by the protein misfolding cyclic amplification (PMCA) technique, which amplifies disease-associated prion protein but does not detect *bona fide* prion infectivity. Here, we demonstrate that *Drosophila* transgenic for bovine prion protein (PrP) expression can serve as a model system for the detection of bovine prions significantly more efficiently than either the mouse prion bioassay or PMCA. Strikingly, bovine PrP transgenic *Drosophila* could detect bovine prion infectivity in the region of a 10^−12^ dilution of classical bovine spongiform encephalopathy (BSE) inoculum, which is 10^6^-fold more sensitive than that achieved by the bovine PrP mouse bioassay. A similar level of sensitivity was observed in the detection of H-type and L-type atypical BSE and sheep-passaged BSE by bovine PrP transgenic *Drosophila*. Bioassays of bovine prions in *Drosophila* were performed within 7 weeks, whereas the mouse prion bioassay required at least a year to assess the same inoculum. In addition, bovine PrP transgenic *Drosophila* could detect classical BSE at a level 10^5^-fold lower than that achieved by PMCA. These data show that PrP transgenic *Drosophila* represent a new tractable prion bioassay for the efficient and sensitive detection of mammalian prions, including those of known zoonotic potential.

Prion diseases are transmissible chronic neurodegenerative central nervous system disorders of humans and various vertebrate species (1). This group of invariably fatal conditions includes Creutzfeldt-Jakob disease (CJD) of humans, bovine spongiform encephalopathy (BSE) of cattle, scrapie of sheep, and chronic wasting disease of cervids. Central to prion diseases is the accumulation of PrP^Sc^, an abnormal conformer of the host protein PrP^C^, in the brains of affected individuals (2). Substantial evidence suggests that the transmissible prion agent comprises principally, if not solely, of proteinaceous material in the form of PrP^Sc^ (3, 4, 5).

Animal prion diseases are a significant public health risk through their potential for zoonotic transmission (6). This risk was realized with the epizootic of classical BSE in UK cattle that was followed by the subsequent emergence of variant CJD (vCJD) in humans and the awareness that these two prion diseases were caused by the same prion strain (7, 8). This highlights the need to maintain surveillance for animal prions in those species destined for human consumption to protect human health. This is particularly relevant because two additional types of bovine prion disease, with unknown zoonotic potential, have been identified. These atypical forms of BSE are characterized by distinct PrP^Sc^ molecular mass profiles, which are either heavy type (H-type) or light type (L-type, or BASE) in comparison with classical BSE (9, 10, 11). Prions lack a nucleic acid–based genome and are not detected by commonly used techniques that identify conventional pathogens, such as viruses and bacteria. Instead, *in vitro* amplification of PrP^Sc^, mediated by prion-seeding activity, a surrogate marker of the actual infectious prion, can be achieved by protein misfolding cyclic amplification (PMCA) (12) and real-time quaking-induced conversion (RT-QuIC) (13). However, the level of PrP^Sc^ and prion infectivity is not always congruent (14, 15), and the only reliable method to detect infectious prions is by bioassay in a suitable experimental host, which has commonly been mice.

The RIII mouse line was used extensively to detect bovine prions in classical BSE pathogenesis studies in cattle but showed relative sensitivity because of the prion transmission barrier between cattle and WT mice. To circumvent this, bovine prion protein (PrP) transgenic mice have been generated that provide facilitated transmission of BSE prions (16). Mice generated by random insertion of multiple copies of a bovine PrP transgene in a murine PrP-ablated background show 100% attack rate for classical BSE prions and display reduced or comparable incubation times compared with that seen in WT mice (17, 18, 19). In contrast, bovine PrP transgenic mice generated by knock-in gene replacement with resultant physiological PrP expression levels show less than 100% attack rate for bovine prions and display extended incubation times for the onset of clinical disease (20, 21). Bovine PrP transgenic mice have collectively been of immense value in the determination of prion infectivity titers in tissues derived from BSE-affected cattle (22). However, even the use of bovine PrP transgenic mice to bioassay BSE prions is cumbersome, time-consuming, and insensitive, especially with low-dose inoculum. For these reasons, it is important to develop more efficient bioassays to assess bovine prions and other species forms of mammalian prion infectivity.

In pursuit of this goal, we have established *Drosophila* as a new host to bioassay mammalian prions (23). Here, we demonstrate that bovine PrP *Drosophila* can detect classical BSE, H-type and L-type atypical BSE, and sheep-passaged BSE at levels 10^6^-fold lower than that achieved by bovine PrP mice. Strikingly, bovine PrP *Drosophila* could detect classical BSE-infected bovine brain homogenate at a dilution as low as 10^−12^. Bioassay of bovine prions in the fly could be performed within 7 weeks, in contrast to the mouse prion bioassay that required at least 1 year to assess the same inocula. In addition, bovine PrP *Drosophila* could detect classical BSE at a level 10^5^-fold lower than *in vitro* PMCA. These data show that PrP *Drosophila* can provide a sensitive, efficient, and economical system for use in the assessment of the risk that zoonotic prions present to human food safety. This in turn provides proof-of-principle that PrP *Drosophila* can contribute to the assessment of new reservoirs of other species forms of potentially zoonotic prions.

## Results

### Propagation of prion-seeding activity in BSE-exposed bovine PrP *Drosophila*

We determined the susceptibility of *Drosophila* to bovine prions by exposure of flies at the larval stage to classical BSE-infected or prion-free bovine brain homogenate. Once hatched, *Drosophila* were transferred to prion-free fly culture tubes. At designated time points during their adult life span, groups of *Drosophila* were euthanized and decapitated, and the homogenate was prepared from the isolated fly heads. These homogenates were used to seed *in vitro* PMCA reactions to detect classical BSE prion-seeding activity. The data in Table 1 show that no prion-seeding activity was detected in PMCA reactions seeded with fly head homogenate prepared from mock-infected bovine PrP *Drosophila*, or from classical BSE-exposed or mock-infected 51D control flies. Similarly, no prion-seeding activity was detected in the head homogenate prepared from 5- or 10-day-old bovine PrP *Drosophila* that had been exposed to classical BSE at the larval stage. However, prion-seeding activity was detected in the head homogenate prepared from similarly treated classical BSE-infected bovine PrP *Drosophila* aged 20, 30, or 40 days.

**Table 1:** Prion-seeding activity in classical BSE-exposed bovine PrP *Drosophila*

Fly line	Inoculum at larval stage	Age (in days) of adult *Drosophila* assessed by PMCA				
		5	10	20	30	40
Bovine PrP	Classical BSE	−	−	+	+	+
	Control	−	−	−	−	−
51D control	Classical BSE	−	−	−	−	−
	Control	−	−	−	−	−

*Elav*x bovine PrP and *Elav* x 51D control *Drosophila* were exposed at the larval stage to classical BSE-infected or prion-free control bovine brain material. At various times after hatching, the head homogenate was prepared from harvested flies and used as seed in PMCA reactions. Positive (+) and negative (−) PMCA reactions shown.

We then assessed whether the prion-seeding activity that accumulates in classical BSE-exposed bovine PrP *Drosophila* could be serially propagated. For this purpose, the head homogenate prepared from 5- and 40-day-old adult *Drosophila* (first-passage flies) was used to inoculate fresh bovine PrP *Drosophila* (second-passage flies) at the larval stage. Second-passage bovine PrP *Drosophila* were allowed to hatch, and groups of flies were euthanized at 5, 30, and 40 days of age when the head homogenate was subjected to PMCA and reaction products analyzed by Western blot for the presence of proteinase K (PK)-resistant PrP27-30.

**Figure 1:**
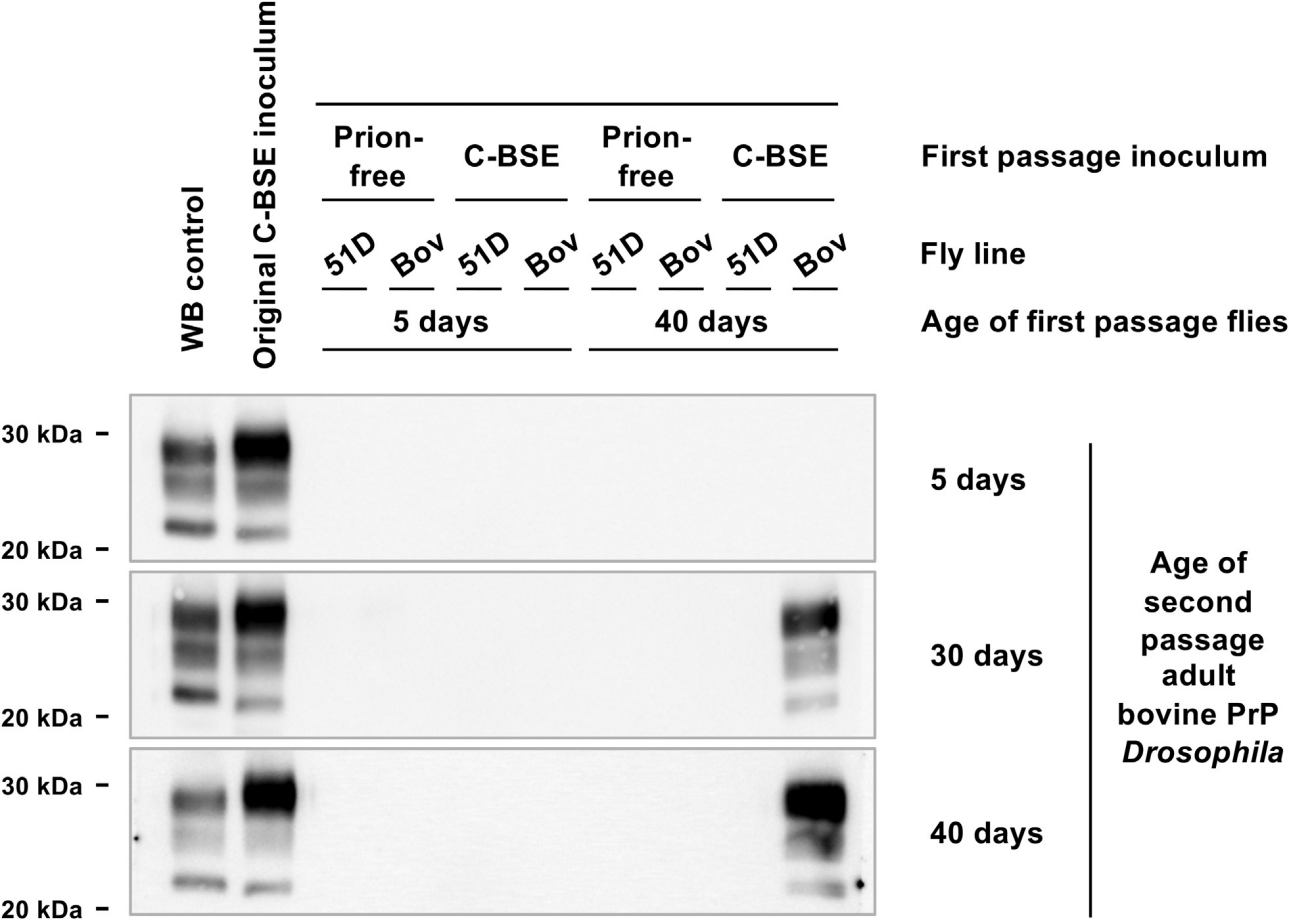
**Serial transmission of prion-seeding activity in classical BSE-exposed bovine PrP *Drosophila*.***Elav* x bovine PrP (Bov) or *Elav* x 51D (51D) *Drosophila* were exposed to classical BSE-infected or prion-free control bovine brain material at the larval stage. The head homogenate was prepared from 5- and 40-day-old adult *Drosophila* (first-passage flies) and used to inoculate fresh *Elav* x bovine PrP *Drosophila* (second-passage flies) at the larval stage. At various times after hatching, the head homogenate was prepared from harvested secondary-passage *Elav* x bovine PrP *Drosophila* and used as seed in PMCA reactions. Western blot was used to detect PK-resistant PrP27-30 in PMCA reaction products. Molecular mass markers in kilodalton are shown on the left. WB control denotes the Western blot control comprising PG127 scrapie-infected sheep brain material included to highlight the low-molecular-weight band of unglycosylated BSE PK-resistant PrP^Sc^. BSE, bovine spongiform encephalopathy; C-BSE, classical BSE; PMCA, protein misfolding cyclic amplification.

The data in Figure 1 show that no PMCA prion-seeding activity was detected in bovine PrP *Drosophila* exposed to the head homogenate from first-passage mock-infected bovine PrP *Drosophila*, or first-passage classical BSE- or mock-infected 51D control flies. However, prion-seeding activity was detected in second-passage bovine PrP *Drosophila* exposed to 40-day-old, but not 5-day-old, head homogenate from first-passage classical BSE-exposed bovine PrP *Drosophila*, when the second passage flies were harvested at 30 or 40 days after inoculation. The molecular profile of PMCA-derived PK-resistant PrP27-30 was typical of classical BSE amplified by PMCA in ovine A^136^R^154^Q^171^ (ARQ) PrP substrate as shown by a lower molecular weight band of unglycosylated PrP compared with that of ovine scrapie material. To show that serial transmission of prion seeding was not unique to classical BSE prions, this experiment was repeated using sheep-passaged BSE inoculum at primary passage in *Drosophila*. Fig. S1 shows that prion-seeding activity was subsequently detected in second-passage bovine PrP *Drosophila* exposed to 40-day-old head homogenate from first-passage ovine BSE-exposed bovine PrP *Drosophila*, when the second-passage flies were harvested at 30 or 40 days after inoculation, but not from 5-day-old flies or similarly treated control flies.

Collectively, these data are consistent with the propagation of bovine prions in bovine PrP *Drosophila*, a view supported by our previous observations that show authentic replication of ovine prions in ovine PrP *Drosophila* (23).

### Level of sensitivity of classical BSE prion detection by bovine PrP *Drosophila*

**Figure 2:**
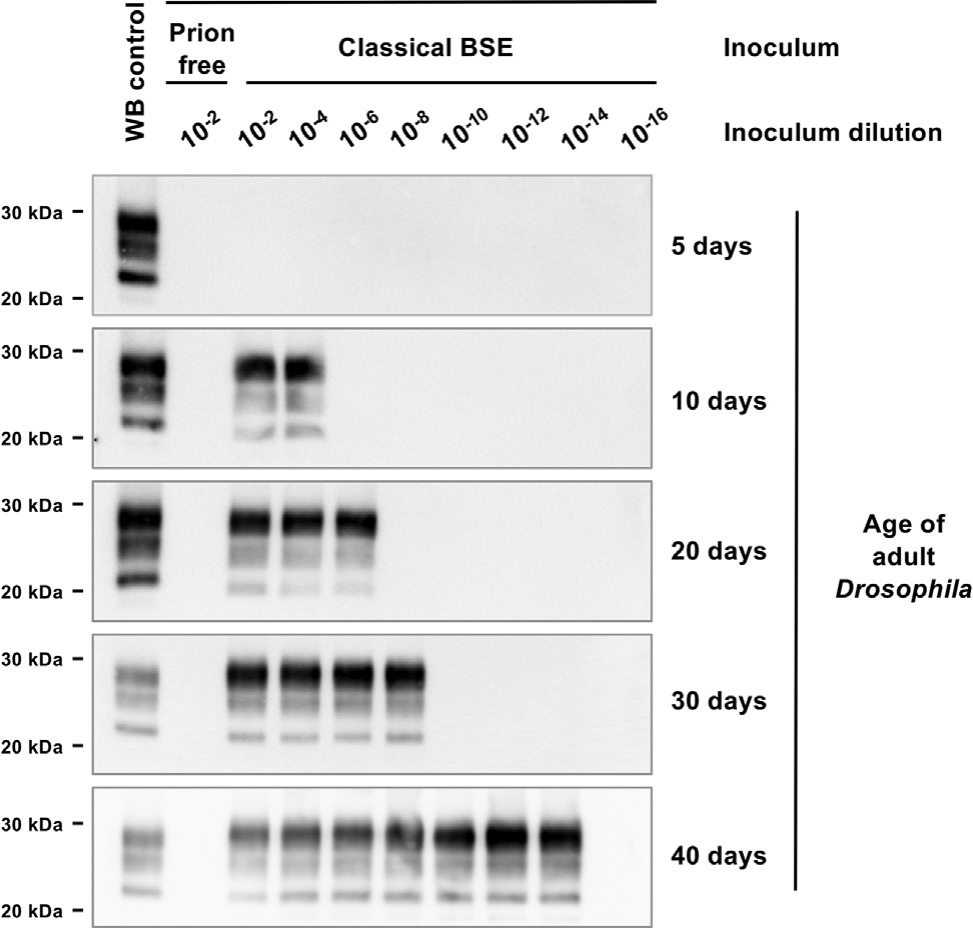
**Sensitivity of prion-seeding activity detection in classical BSE-exposed bovine PrP *Drosophila*.** Adult *Elav* x bovine PrP *Drosophila* were assessed for prion-seeding activity after exposure at the larval stage to 10^−2^ to 10^−20^ dilutions of classical BSE-infected bovine brain material. The control inoculum was a 10^−2^ dilution of prion-free bovine brain homogenate. At various times after hatching, the head homogenate was prepared from harvested bovine PrP *Drosophila* and used as seed in PMCA reactions. Western blot was used to detect PK-resistant PrP27-30 in PMCA reaction products seeded with the head homogenate from *Drosophila* exposed to classical BSE-infected or control bovine brain homogenate. (Data for adult *Elav* x bovine PrP *Drosophila* exposed to 10^−2^ to 10^−16^ dilutions of classical BSE-infected bovine brain material are shown). Molecular mass markers in kilodalton are shown on the left. WB control denotes Western blot control comprising PG127 scrapie-infected sheep brain material included to highlight the low-molecular-weight band of unglycosylated BSE PK-resistant PrP27-30. BSE, bovine spongiform encephalopathy; PK, proteinase K; PMCA, protein misfolding cyclic amplification.

We next established the level of sensitivity of classical BSE prion detection by bovine PrP *Drosophila*. We first used PMCA followed by Western blot detection of PK-resistant PrP27-30 to reveal prion-seeding activity in adult *Drosophila* exposed to dilutions of classical BSE inoculum at the larval stage. Fig. S2 shows that while prion-seeding activity was detected in the head homogenate prepared from classical BSE-exposed bovine PrP *Drosophila* aged ≥20 days, the sensitivity of detection progressively increased as the flies aged. For example, prion-seeding activity was detected in the head homogenate of 20-day-old adult *Drosophila* after exposure at the larval stage to ≤10^−8^ dilutions of classical BSE-infected bovine brain material, while 40-day-old flies were PMCA-positive after exposure to ≤10^−14^ dilutions of the same inoculum. In this particular experiment, the highest dilution of classical BSE tested was 10^−14^ dilution of inoculum. To determine the end-point of titration, we repeated the experiment using fresh dilutions of classical BSE, in the range 10^−2^ to 10^−20^ dilutions of inoculum, and fresh bovine PrP *Drosophila*. The data in Figure 2 show that prion-seeding activity was detected with increasing sensitivity as the flies aged and that the end point of titration was 10^−14^ dilution of inoculum.

We next showed that the sensitivity of bovine PrP *Drosophila* for classical BSE prions determined by detection of prion-seeding activity correlated with the level of prion-induced neurotoxic phenotype seen in these flies, which was assessed by negative geotaxis climbing assay or survival.

**Figure 3:**
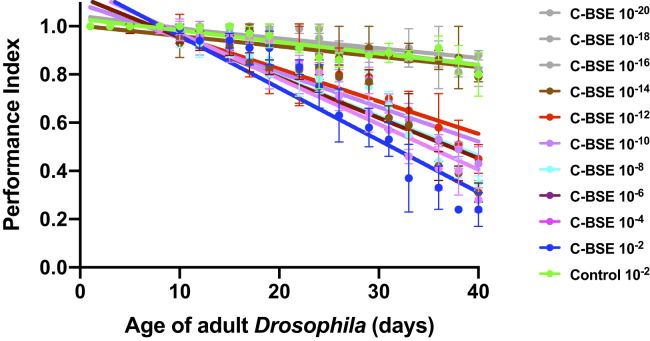
**Accelerated loss of locomotor ability in classical BSE-exposed bovine PrP *Drosophila*.** Adult *Elav* x bovine PrP *Drosophila* were assessed for their locomotor ability by a negative-geotaxis climbing assay after exposure at the larval stage to 10^−2^ to 10^−20^ dilutions of classical BSE-infected bovine brain material. Control inoculum was a 10^−2^ dilution of prion-free bovine brain material (control 10^−2^). The data shown are linear regression plots of the mean performance index ± SD for three groups of flies per time point calculated as described in Experimental procedures. Statistically significant responses were demonstrated between days 15 and 40 of the locomotor ability assay when assessed by one-way ANOVA (with Dunnett’s multiple comparisons test) for classical BSE dilutions 10^−2^ to 10^−10^*versus* prion-free control bovine brain material (*p* ≤ 0.029) and unpaired Student’s *t* test (two tailed) for classical BSE diluted 10^−12^*versus* prion-free control bovine brain material (*p* = 0.0016). BSE, bovine spongiform encephalopathy; C-BSE, classical BSE.

Fig. S3 shows that adult bovine PrP *Drosophila*, exposed to classical BSE at the larval stage, developed an accelerated decrease in climbing ability compared with control-treated flies. The toxic phenotype, which became progressively more severe with age, decreased with increasing dilution of classical BSE inoculum to which bovine PrP *Drosophila* were exposed at the larval stage. Fig. S3 also shows there was no difference in the climbing ability between 51D control flies exposed to classical BSE prion infectivity or control prion-free bovine brain homogenate. In this experiment, the highest dilution of classical BSE inoculum tested was 10^−14^ dilution of inoculum. We repeated the experiment using fresh dilutions of classical BSE, in the range 10^−2^ to 10^−20^ dilutions of inoculum, and fresh bovine PrP *Drosophila* to determine the end point of titration. The data in Figure 3 show the end point of titration in this experiment was 10^−12^ dilution of classical BSE inoculum.

**Figure 4:**
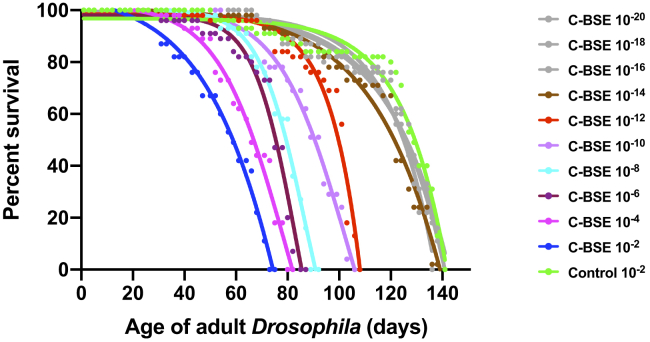
**Accelerated loss of survival of classical BSE-exposed bovine PrP *Drosophila*.** Adult *Elav* x bovine PrP *Drosophila* were assessed for survival after exposure at the larval stage to 10^−2^ to 10^−20^ dilutions of classical BSE-infected bovine brain material. The control inoculum was a 10^−2^ dilution of prion-free bovine brain material (control 10^−2^). The number of surviving flies was recorded three times a week as described in Experimental procedures and the data shown as Kaplan–Meier plots. BSE, bovine spongiform encephalopathy; C-BSE, classical BSE.

The data in Figure 4 show that adult bovine PrP *Drosophila*, previously exposed to classical BSE at the larval stage, displayed an accelerated loss of survival compared with control-treated flies. Table S1 shows that the median survival time of bovine PrP *Drosophila* exposed to control prion-free inoculum was 127 days. In contrast, exposure of bovine PrP *Drosophila* larvae to a 10^−2^ dilution of classical BSE-infected bovine brain homogenate resulted in a median survival time of 59 days for adult flies, which progressively lengthened upon exposure to increasing dilution of classical BSE inoculum at the larval stage. Accelerated loss of survival by adult bovine PrP *Drosophila* was observed after exposure of larvae to dilutions in the range 10^−2^ to 10^−12^ dilution of classical BSE-infected bovine brain homogenate.

Collectively, the data from these different assays show that the limit of sensitivity of bovine PrP *Drosophila* for detection of classical BSE bovine prion infectivity was in the range of 10^−12^ to 10^−14^ dilution of infected bovine brain material.

### Detection of atypical BSE and ovine BSE prions by bovine PrP *Drosophila*

We subsequently determined the ability of bovine PrP *Drosophila* to detect atypical BSE and ovine-passaged BSE prion infectivity. To do so, we performed negative geotaxis climbing assays using adult bovine PrP *Drosophila* previously exposed at the larval stage to dilutions of these various BSE inocula.

**Figure 5:**
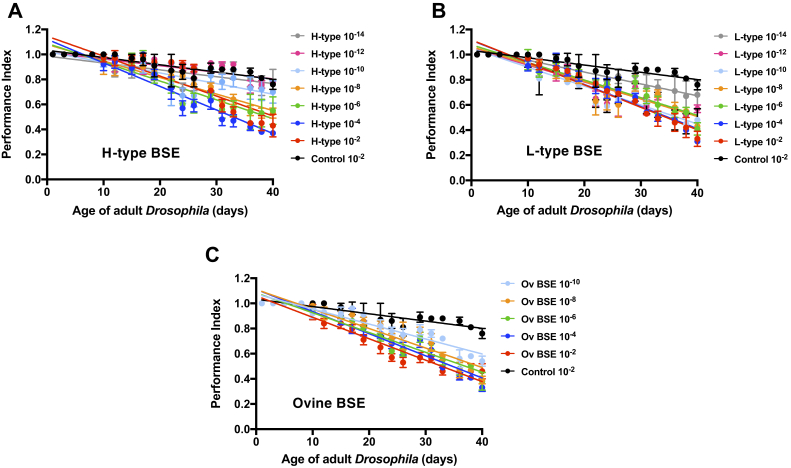
**Accelerated loss of locomotor ability in H- or L-type atypical BSE- or ovine BSE-exposed bovine PrP *Drosophila*.** Adult *Elav* x bovine PrP *Drosophila* were assessed for their locomotor ability by a negative-geotaxis climbing assay after exposure at the larval stage to the dilutions shown of (*A*) H-type BSE; (*B*) L-type BSE; or (*C*) ovine BSE inoculum. The control inoculum was a 10^−2^ dilution of prion-free bovine brain material (control 10^−2^). The data shown are linear regression plots of the mean performance index ± SD for three groups of flies per time point calculated as described in Experimental procedures.

The data in Figure 5 demonstrate that adult bovine PrP transgenic *Drosophila* showed an accelerated decline in locomotor activity after exposure at the larval stage to atypical H-type BSE (Fig. 5*A*), L-type BSE (Fig. 5*B*), ovine-passaged BSE (Fig. 5*C*) compared with similar flies exposed to control prion-free inoculum. As expected, the rate of decline of locomotor activity diminished gradually with increasing dilution of prion inoculum. Bovine PrP *Drosophila* were responsive to dilutions of H-type BSE and ovine-passaged BSE in the range of 10^−2^ to 10^−10^ dilution of the prion-infected bovine brain homogenate, and 10^−2^ to 10^−14^ dilution of L-type BSE-infected bovine brain homogenate. Fig. S4 shows that there was no difference in the climbing ability between 51D control flies exposed to atypical BSE and ovine-passaged BSE compared with control prion-free brain material.

### Detection of bovine prions by PMCA and bioassay in mice

We compared the sensitivity of our invertebrate-based prion bioassay to detect bovine prions to that of *in vitro* PMCA and to the mouse prion bioassay.

We first used PMCA to end-point titrate the same classical BSE inoculum used to infect bovine PrP *Drosophila*. A dilution series of the classical BSE isolate was used as seed in PMCA reactions that were subsequently tested for the presence of PK-resistant PrP^Sc^. The data in Table 2 show that positive PMCA activity was observed in reaction tubes seeded with dilutions ≤10^−7^ dilution of the classical BSE isolate.

**Table 2:** End-point titration of classical BSE inoculum by *in vitro* PMCA

Classical BSE dilution	PMCA-positive reactions
10^−4^	6/6
10^−5^	6/6
10^−6^	6/6
10^−7^	3/6
10^−8^	0/6
10^−9^	0/6

A 10-fold dilution series of classical BSE-infected bovine brain homogenate was subjected to PMCA (six replicate wells per dilution) as described in Experimental procedures using brain material from transgenic mice overexpressing the A^136^R^154^Q^171^ (ARQ) variant of the sheep prion protein. Reactions were subjected to up to four amplification rounds. After each round, reaction products (one volume) were mixed with fresh substrate (nine volumes) to seed the following round. PMCA reaction products were analyzed by dot blot/Western blot for the presence of PK-resistant PrP27-30. The number of PK-resistant PrP^Sc^ Western blot–positive reactions/total number of reactions is reported.

A second dilution series of the classical BSE inoculum was prepared and inoculated by the intracerebral route into tg110 bovine PrP transgenic mice (18). The data in Table 3 show that positive transmission, based on PrP^Sc^ accumulation in the brain of inoculated mice (see Fig. S5), was observed in animals exposed to ≤10^−4^ dilutions of the classical BSE isolate. Table 3 also shows that tg110 mice developed prion disease after challenge with the H- and L-type BSE and ovine-passaged BSE with a similar sensitivity to that seen for classical BSE. A collective feature of these mouse prion bioassays was the long duration, which was extended with increasing dilutions of the inoculum.

**Table 3:** Transmission of BSE isolates in tg110 bovine PrP mice

Inoculum dilution	BSE inoculum							
	Classical BSE		H-BSE		L-BSE		Ovine BSE	
	Attack rate	Survival time	Attack rate	Survival time	Attack rate	Survival time	Attack rate	Survival time
10^−1^	10/10	228 ± 16	10/10	249 ± 26	6/6	179 ± 12	10/10	252 ± 25
10^−2^	10/10	259 ± 22	7/8	261 ± 43	9/10	200 ± 23	9/10	246 ± 26
10^−4^	10/10	332 ± 38	6/9	360 ± 56	10/10	261 ± 26	10/10	361 ± 50
10^−6^	0/10	471 ± 140	0/10	407 ± 95	1/8	274	0/6	410 ± 130
10^−8^	0/9	574 ± 92	0/10	395 ± 115	0/10	403 ± 113	0/10	434 ± 76

A dilution series (v/v) of bovine and ovine BSE isolates were each injected into the bovine PrP mouse line tg110 (*n* = 10 mice per inoculum) intracerebrally. Mice were monitored for the development of clinical signs of mouse prion disease and euthanized at the time of appearance of terminal signs of disease. Survival was measured as the time (in days ± SD) between inoculation and death. Prion disease was confirmed by detection of disease-associated PrP using immunohistochemistry. For each group of mice, the attack rate was calculated as the number of mice diagnosed as prion positive over the total number of live mice at the time when the first prion-positive animal was identified. Representative IHC data are shown in Fig. S5.

## Discussion

Here, we show that *Drosophila* transgenic for bovine PrP are highly susceptible to BSE prion infectivity. Strikingly, we have shown that bovine PrP *Drosophila* were significantly more sensitive than bovine PrP transgenic mice and PMCA for the detection of BSE prions. Our studies demonstrated that classical BSE-exposed bovine PrP *Drosophila* displayed core features of mammalian prion disease, namely progressive accumulation of transmissible prion-seeding activity accompanied by the increasing severity of a neurotoxic phenotype, evident as impaired locomotor ability and accelerated loss of survival. In addition, PMCA products from reactions seeded with classical BSE-exposed bovine PrP *Drosophila* head homogenate displayed PK-resistant PrP27-30 characteristic of this form of BSE. Collectively, these observations were indicative of *bona fide* bovine prion infection of *Drosophila*, a normally PrP-null insect host, rendered transgenic solely for bovine PrP. This view is supported by our previous observations that have demonstrated that PrP transgenic *Drosophila* are permissive for the authentic replication of mammalian prions (23). Furthermore, our observations reported here demonstrate for the first time the efficient ability of an invertebrate host to bioassay zoonotic mammalian prions.

Historically, the in-bred mouse line RIII was used to detect classical BSE prion infectivity (24). A significant limitation of this experimental host was its reduced sensitivity to low-titer inoculum because bovine prions have to cross the mouse-to-cattle species barrier to propagate. The development of mice transgenic for bovine PrP, which allowed the species barrier to be circumvented, was found to be more than 10^4^-fold more sensitive than RIII mice for the detection of classical BSE (22). Here, we have shown that bovine PrP *Drosophila*, assessed either biochemically or phenotypically, could detect as low as 10^−12^ dilution of classical BSE inoculum, which was significantly more sensitive than bioassay in tg110 bovine PrP transgenic mice and by *in vitro* PMCA. Bovine PrP *Drosophila* also showed a similar enhanced sensitivity compared with tg110 mice with regard to detection of H- and L-type bovine BSE, and ovine-passaged BSE.

The high sensitivity shown by bovine PrP *Drosophila* for classical BSE shows that low levels of prion infectivity can be detected by the bioassay in this invertebrate host. The *in vitro*–based techniques of PMCA and RT-QuIC, which both rely on the biochemical detection of prion-seeding activity, have shown that vCJD prion-infected human brain material can be detected when diluted out to 10^−12^ and 10^−14^, respectively (12, 13). Collectively, these observations provide a robust correlation for the presence of infectious prion particles and detectable levels of disease-associated PrP in highly dilute samples of mammalian prion inocula. The high level of sensitivity of PrP transgenic *Drosophila* toward mammalian prions is not restricted to classical BSE because we have previously reported that ovine PrP transgenic *Drosophila* are more sensitive to sheep scrapie prions than ovine PrP transgenic mice (25). These transmission studies of the same prion inoculum in *Drosophila* and mice, transgenic for the same species form of PrP, infer that prion titers are host specific, which has implications for how an infectious prion particle is defined. It is hypothesized that transmissible prions comprise an ensemble of PrP^Sc^ conformers (26). An extension of this view is that different misfolded PrP structures each have a different infectious potential that only becomes evident upon passage in an appropriate host.

Several possible explanations exist for the increased sensitivity to mammalian prions shown by *Drosophila* in comparison to mice: a broader range of PrP^Sc^ conformers may be infectious in *Drosophila* than the mouse; *Drosophila* may provide a more favorable environment for prion replication, accumulation, and spread than mice, either through enhanced prion formation and/or reduced prion clearance or both of these effects; the exposure of *Drosophila* to prions at an earlier stage of development compared with that of mice may fortuitously render the invertebrate host more sensitive to prion replication and prion-induced toxicity during adulthood. Any of these possibilities may be underpinned by glycosylation, which is an important factor in the determination and maintenance of conformation, function, and interactions of glycoproteins (27, 28). Mammalian N-linked carbohydrate moieties, including those attached to PrP^C^ expressed in the natural host, typically comprise complex glycans that consist of N-acetylglucosamine, mannose, galactose, and terminal sialic acid residues (29, 30, 31). In contrast, the majority of neurons in the *Drosophila* brain synthesize N-linked glycans with core structures similar or identical to those produced by all eukaryotes but fail to acquire complex carbohydrate structures (32, 33, 34). It has been reported that *in vitro* PMCA using de-sialylated PrP^C^ as the substrate can proceed with a 10- to 10,000-fold enhanced amplification rate compared with that seen with normal PrP^C^ (35). In addition, the use of de-sialylated PrP^C^ led to a mitigation of the species barrier during *in vitro* PMCA. These observations suggest that sialic acid residues play an important role in the control of PrP^C^ to PrP^Sc^ conversion and have a negative impact upon this process. In this context, the low sialylation status of proteins in *Drosophila* could lower the energy barrier for prion formation and thereby provide a favorable environment for successful infection of this host with mammalian prions, which is of particular importance with low-dose inoculum.

The epizootic of classical BSE in UK cattle and the subsequent emergence of vCJD in humans (7, 8) have highlighted the threat animal prion diseases present to human food safety (36). Strict controls protect the human food chain from classical BSE, including the removal from all cattle carcasses destined for human consumption of those tissues most likely to contain BSE prion infectivity at the time cattle are slaughtered. These tissues, known as specified risk material, do not enter the human food chain. Specified risk materials have been defined using tissues from cattle experimentally infected with classical BSE (37, 38) by detection of PrP^Sc^ and prion infectivity levels to determine their relative levels of risk. The identification of atypical BSE poses a new challenge to human food safety because their zoonotic potential is unknown. However, experimental transmissions of atypical BSE sources indicate that they can give rise to either classical BSE or novel strains whose zoonotic potential is undetermined (39, 40, 41). Ideally, new cattle pathogenesis studies are required to determine if current protocols designed for classical BSE detection would also be sufficient to prevent atypical BSE from entering the food chain.

Our studies reported here have demonstrated that bovine PrP *Drosophila* provide a sensitive and robust bioassay to assess the risk that BSE prions present to human food safety. This in turn provides proof-of-principle that PrP *Drosophila* can contribute to the analysis of new reservoirs of other species forms of potentially zoonotic prions, such as those from prion-diseased cervids that have been detected in Europe (42), or camels for which no transgenic mice have yet been developed (43). The ability to produce transgenic *Drosophila* models which express mammalian PrP in a matter of months in contrast to years required to produce transgenic mice shows that this invertebrate host is of significant value in contributing to the detection of prion infectivity for novel and emerging animal prion diseases.

## Experimental procedures

### Generation of bovine PrP transgenic *Drosophila*

*Drosophila* transgenic for bovine PrP (six octapeptide repeats) were generated by pUASTattB/PhiC31–mediated site-specific transformation (44). The bovine PrP transgene comprised DNA encoding an insect secretion signal peptide at the 5′ end (45) followed by DNA encoding mature bovine PrP (GenBank accession number AF455119) (amino acid residues 25–232) and DNA encoding the bovine PrP glycosylphosphatidylinositol anchor signal sequence (amino acid residues 233–264) at the 3′ end. The bovine PrP transgene inserted into the *Drosophila* genome was prepared by a two-step PCR. The first PCR used plasmid DNA that contained bovine PrP DNA as the substrate and oligonucleotide primers PD1F: 5′-GTC CAT CTT CTG GCT GCT CAG ACC TTC GCC CAG AAG AAG CGA CCA AAA CCT GG-3′ and Bov PD1R: 5′-GGG GAA GAG AAG AGG ATC ACA CTT GCC CCT CGT TGG TAA TAA GCC TGG GAT TCT CT-3′ in the presence of *Pfu* DNA polymerase (Promega). The PCR conditions consisted of an initial denaturation at 95 °C for 2 min followed by 30 cycles of denaturation at 95 °C for 30 s, primer annealing at 55 °C for 30 s and extension at 72 °C for 1 min, and a final extension of PCR products at 72 °C for 10 min. A second PCR was carried out using the 627-bp product of the first PCR as the substrate and oligonucleotide primers PD2F: 5′-GGC GAA TTC ATG GCG AGC AAA GTC TCG ATC CTT CTC CTG CTA ACC GTC CAT CTT CTG C-3′ and Bov PD2R: 5′-GTC CGC TCG AGC TAT CCT ACT ATG AGA AAA ATG AGG AAA GAG ATG AGG AGG ATC ACA GGA GGG GAA GAG AAG AGG-3′. The reaction conditions for this second PCR were the same as for the first apart from the primer annealing temperature which was 64 °C for 30 s. The PCR primers PD2F and Bov PD2R contained *EcoR1* and *Xho1* restriction sites, respectively, that allowed directional cloning of the 788-bp PCR product into the *Drosophila* transgenesis vector pUASTattB. Primer PD2R contained a stop codon ahead of the *Xho1* restriction site. The bovine PrP transgene was subsequently ligated into the transgenesis vector pUASTattB and rescued by transformation in DH5α bacteria. pUASTattB-Bovine PrP plasmid DNA was isolated from transformed bacteria by an alkaline lysis method using the Qiagen maxiprep kit and the PrP construct insert verified by DNA sequence analysis. Site-specific transformation of the pUASTattB-PrP constructs into the RFP-free 51D variant fly line (y[1] M{vas-int.Dm}ZH-2A w[∗]; M{3xP3-RFP.attP}ZH-51D) was performed by the Department of Genetics, Cambridge University. F1 flies were balanced, the inserted PrP transgene verified by DNA sequence analysis, and a viable fly line w; M{Bov-PrP(GPI).attP}ZH-51D maintained as a balanced stock by conventional fly crosses. The following fly lines were obtained from the Department of Genetics, University of Cambridge, UK. *Elav-GAL4* (P{w[+mW.hs]=GawB}elav[C155]), 51D (w; M{3xP3-RFP.attP}ZH-51D). All fly lines were raised on standard cornmeal media at 25 °C and maintained at low to medium density. Flies were used in the assays described below or harvested at various time points and then frozen at −80 °C until required.

### Preparation of *Drosophila* head homogenate

Whole flies in an Eppendorf tube were frozen in liquid nitrogen for 10 min and then vortexed for 2 min to cause decapitation. Individual fly heads were isolated and placed in clean Eppendorf tubes using a fine paint brush. PBS, pH 7.4, was added to give 1 μl per head (original fly brain homogenate), and homogenates were prepared by manual grinding of the fly heads with sterilized plastic pestles.

### Prion inocula

BSE inocula consisted of the brain homogenate, prepared in normal saline, of the cerebral cortex tissue from confirmed cases of bovine classical BSE (PG0045/90), atypical H-type BSE (PG1129/10), atypical L-type BSE (PG1345/10), and sheep-passaged BSE (PG1834/04). Confirmed BSE-free bovine brain tissue (PG1477/08) was used as the control material. All isolates were obtained from the APHA.

### Prion inoculation of *Drosophila*

#### Primary transmission of cattle BSE to fly

*Drosophila* at the larval stage of development were exposed to the BSE-infected or prion-free control bovine brain homogenate. Two hundred and fifty microliters of 1% (w/v) of the bovine brain homogenate, or a 1/100 dilution series (v/v) of these samples, prepared in PBS, pH 7.4, was added to the top of the cornmeal that contained third instar *Drosophila* larvae in 3-inch plastic vials. After eclosion (*i.e.*, hatching), flies were transferred to fresh nontreated vials.

#### Secondary transmission of fly-passaged BSE to fly

*Drosophila* head homogenates were prepared from 5- or 40-day-old flies that had been exposed at the larval stage to classical BSE-positive or prion-free control bovine brain material. Two hundred and fifty microliters of a 10% (v/v) dilution of the original fly brain homogenate (prepared as described above) was added to the top of the cornmeal that contained third instar *Drosophila* larvae in 3-inch plastic vials. Flies were transferred to fresh, nontreated vials after eclosion.

### PMCA

PMCA was carried out as previously described (46). The substrate consisted of 10% (w/v) tgShpXI (47) (A^136^R^154^Q^171^ ovine PrP) transgenic mouse brain homogenate in PBS, pH 7.4, 0.1% Triton X-100, and 150 mM NaCl buffer. Five microliters of the fly head homogenate was mixed with 45 μl of the substrate in 0.2-ml thin-wall PCR tubes. Sealed tubes were then placed in the horn of a Misonix 4000 sonicator for one round of 96 cycles. Each cycle consisted of a 10-s sonication step (70% of power) followed by a 14-min and 50-s incubation step. Twenty microliters of each reaction mix was subsequently treated with PK (4 μg of PK per milligram of protein) at 37 °C for 2 h and the reaction stopped by adding Pefabloc (4 mM final concentration). PK-resistant PrP^Sc^ was detected by Western blot as previously described (46) using mAb Sha31 (48).

### Negative-geotaxis climbing assay

The locomotor ability of flies was assessed in a negative-geotaxis climbing assay initiated with 45 (3 × n = 15) age-matched, premated female flies in each treatment group (49). *Drosophila* were placed in adapted plastic 25-ml pipettes that were used as vertical climbing columns and allowed to acclimatize for 30 min before assessment of their locomotor ability. Flies were tapped to the bottom of the pipette (using the same number and intensity of taps on each occasion) and then allowed to climb for 45 s. At the end of the climbing period, the number of flies above the 25-ml mark, the number below the 2 ml mark, and the number in between the 2 ml and 25 ml mark were recorded. This procedure was performed three times at each time point. The performance index (PI) was calculated for each group of 15 flies (average of three trials) using the formula: PI = 0.5 × (*n*total + *n*top − *n*bottom)/*n*total, where *n*total is the total number of flies, *n*top is the total number of flies at the top, and *n*bottom is the total number of flies at the bottom. A PI value of 1 is recorded if all flies climb to the top of the tube, whereas the value is 0 if no flies climb the tube past the 2-ml mark. The mean PI ± SD at individual time points for each treatment group was plotted as a regression line.

### Survival assay

Newly eclosed flies were allowed to mature and mate for 24 h before the females were separated and collected for survival assays. Forty-five flies of each genotype were housed in groups of 15, and the flies were flipped every 2 to 3 days onto fresh food. The number of dead flies was recorded three times a week. Survival curves were calculated using the Kaplan–Meier statistics, and differences between them were analyzed by the log-rank method using Prism (GraphPad Software Inc). Displayed survival curves were plotted with smoothed function.

### Mouse bioassay and immunohistochemistry

After confirmation of sterility by aerobic culture, a 1/10 dilution series (v/v) of bovine and ovine BSE isolates were each injected into the bovine PrP mouse line tg110 (18) (*n* = 10 mice per inoculum) intracerebrally (20 μl). Mice were monitored for the development of clinical signs of mouse prion disease and euthanized at the time of appearance of terminal signs of disease. Survival was measured as the time (in days) between inoculation and death. For each group of mice, survival times are presented as the mean ± SD. Prion disease was confirmed by detection of PrP^Sc^ using immunohistochemistry as described previously (50). For each group of mice, the attack rate was calculated as the number of mice diagnosed as prion positive over the total number of live mice at the time when the first prion-positive animal was identified. Representative immunohistochemistry data are shown in Fig. S5. All regulated procedures involving experimental animals were carried out under a project and personal license authority issued in accordance with The Animals (Scientific Procedures) Act 1986 under Home Office license 70/7167.

### Statistical analysis

Statistical analysis of the negative-geotaxis climbing assay data was performed by one-way ANOVA together with Dunnett’s multiple comparisons test and the unpaired Student’s *t* test (two tailed). Statistical analysis of the median survival times was carried out using Kaplan–Meier plots. All statistical analyses were performed using Prism (GraphPad Software Inc).

## Data availability

All data are contained within the article.

## Supporting information

This article contains supporting information.

## Conflict of interest

The authors declare that they have no conflicts of interest with the contents of this article.

## References

[1] Prusiner S.B. (2004). Prion Biology and Diseases.

[2] Bolton D.C., McKinley M.P., Prusiner S.B. (1982). Identification of a protein that purifies with the scrapie prion. Science.

[3] Prusiner S.B. (1982). Novel proteinaceous infectious particles cause scrapie. Science.

[4] Legname G., Baskakov I.V., Nguyen H.O., Riesner D., Cohen F.E., DeArmond S.J., Prusiner S.B. (2004). Synthetic mammalian prions. Science.

[5] Wang F., Wang X., Yuan C.G., Ma J. (2010). Generating a prion with bacterially expressed recombinant prion protein. Science.

[6] Wells G.A., Scott A.C., Johnson C.T., Gunning R.F., Hancock R.D., Jeffrey M., Dawson M., Bradley R. (1987). A novel progressive spongiform encephalopathy in cattle. Vet. Rec..

[7] Bruce M.E., Will R.G., Ironside J.W., McConnell I., Drummond D., Suttie A., McCardle L., Chree A., Hope J., Birkett C., Cousens S., Fraser H., Bostock C.J. (1997). Transmissions to mice indicate that 'new variant' CJD is caused by the BSE agent. Nature.

[8] Hill A.F., Desbruslais M., Joiner S., Sidle K.C., Gowland I., Collinge J., Doey L.J., Lantos P. (1997). The same prion strain causes vCJD and BSE. Nature.

[9] Biacabe A.G., Laplanche J.L., Ryder S., Baron T. (2004). Distinct molecular phenotypes in bovine prion diseases. EMBO Rep..

[10] Casalone C., Zanusso G., Acutis P., Ferrari S., Capucci L., Tagliavini F., Monaco S., Caramelli M. (2004). Identification of a second bovine amyloidotic spongiform encephalopathy: Molecular similarities with sporadic Creutzfeldt-Jakob disease. Proc. Natl. Acad. Sci. U. S. A..

[11] Jacobs J.G., Langeveld J.P., Biacabe A.G., Acutis P.L., Polak M.P., Gavier-Widen D., Buschmann A., Caramelli M., Casalone C., Mazza M., Groschup M., Erkens J.H., Davidse A., van Zijderveld F.G., Baron T. (2007). Molecular discrimination of atypical bovine spongiform encephalopathy strains from a geographical region spanning a wide area in Europe. J. Clin. Microbiol..

[12] Saborio G.P., Permanne B., Soto C. (2001). Sensitive detection of pathological prion protein by cyclic amplification of protein misfolding. Nature.

[13] Wilham J.M., Orru C.D., Bessen R.A., Atarashi R., Sano K., Race B., Meade-White K.D., Taubner L.M., Timmes A., Caughey B. (2010). Rapid end-point quantitation of prion seeding activity with sensitivity comparable to bioassays. PLoS Pathog..

[14] Lasmezas C.I., Deslys J.P., Robain O., Jaegly A., Beringue V., Peyrin J.M., Fournier J.G., Hauw J.J., Rossier J., Dormont D. (1997). Transmission of the BSE agent to mice in the absence of detectable abnormal prion protein. Science.

[15] Miyazawa K., Emmerling K., Manuelidis L. (2011). High CJD infectivity remains after prion protein is destroyed. J. Cell. Biochem..

[16] Groschup M.H., Buschmann A. (2008). Rodent models for prion diseases. Vet. Res..

[17] Scott M.R., Safar J., Telling G., Nguyen O., Groth D., Torchia M., Koehler R., Tremblay P., Walther D., Cohen F.E., DeArmond S.J., Prusiner S.B. (1997). Identification of a prion protein epitope modulating transmission of bovine spongiform encephalopathy prions to transgenic mice. Proc. Natl. Acad. Sci. U. S. A..

[18] Castilla J., Gutierrez Adan A., Brun A., Pintado B., Ramirez M.A., Parra B., Doyle D., Rogers M., Salguero F.J., Sanchez C., Sanchez-Vizcaino J.M., Torres J.M. (2003). Early detection of PrPres in BSE-infected bovine PrP transgenic mice. Arch. Virol..

[19] Buschmann A., Pfaff E., Reifenberg K., Muller H.M., Groschup M.H. (2000). Detection of cattle-derived BSE prions using transgenic mice overexpressing bovine PrP(C). Arch. Virol. Suppl..

[20] Bishop M.T., Hart P., Aitchison L., Baybutt H.N., Plinston C., Thomson V., Tuzi N.L., Head M.W., Ironside J.W., Will R.G., Manson J.C. (2006). Predicting susceptibility and incubation time of human-to-human transmission of vCJD. Lancet Neurol..

[21] Wilson R., Hart P., Piccardo P., Hunter N., Casalone C., Baron T., Barron R.M. (2012). Bovine PrP expression levels in transgenic mice influence transmission characteristics of atypical bovine spongiform encephalopathy. J. Gen. Virol..

[22] Buschmann A., Groschup M.H. (2005). Highly bovine spongiform encephalopathy-sensitive transgenic mice confirm the essential restriction of infectivity to the nervous system in clinically diseased cattle. J. Infect. Dis..

[23] Thackray A.M., Andreoletti O., Bujdoso R. (2018). Mammalian prion propagation in PrP transgenic Drosophila. Brain.

[24] Wells G.A., Dawson M., Hawkins S.A., Green R.B., Dexter I., Francis M.E., Simmons M.M., Austin A.R., Horigan M.W. (1994). Infectivity in the ileum of cattle challenged orally with bovine spongiform encephalopathy. Vet. Rec..

[25] Thackray A.M., Andreoletti O., Bujdoso R. (2016). Bioassay of prion-infected blood plasma in PrP transgenic Drosophila. Biochem. J..

[26] Collinge J., Clarke A.R. (2007). A general model of prion strains and their pathogenicity. Science.

[27] O'Connor S.E., Imperiali B. (1996). Modulation of protein structure and function by asparagine-linked glycosylation. Chem. Biol..

[28] Helenius A., Aebi M. (2001). Intracellular functions of N-linked glycans. Science.

[29] Endo T., Groth D., Prusiner S.B., Kobata A. (1989). Diversity of oligosaccharide structures linked to asparagines of the scrapie prion protein. Biochemistry.

[30] Stimson E., Hope J., Chong A., Burlingame A.L. (1999). Site-specific characterization of the N-linked glycans of murine prion protein by high-performance liquid chromatography/electrospray mass spectrometry and exoglycosidase digestions. Biochemistry.

[31] Moremen K.W., Tiemeyer M., Nairn A.V. (2012). Vertebrate protein glycosylation: Diversity, synthesis and function. Nat. Rev. Mol. Cell Biol..

[32] März L., Altmann F., Staudacher E., Kubelka V. (1995). Protein Glycosylation in Insects.

[33] Marchal I., Jarvis D.L., Cacan R., Verbert A. (2001). Glycoproteins from insect cells: Sialylated or not?. Biol. Chem..

[34] Repnikova E., Koles K., Nakamura M., Pitts J., Li H., Ambavane A., Zoran M.J., Panin V.M. (2010). Sialyltransferase regulates nervous system function in Drosophila. J. Neurosci..

[35] Katorcha E., Makarava N., Savtchenko R., D'Azzo A., Baskakov I.V. (2014). Sialylation of prion protein controls the rate of prion amplification, the cross-species barrier, the ratio of PrPSc glycoform and prion infectivity. PLoS Pathog..

[36] Zeidler M., Ironside J.W. (2000). The new variant of Creutzfeldt-Jakob disease. Rev. Sci. Tech..

[37] Kaatz M., Fast C., Ziegler U., Balkema-Buschmann A., Hammerschmidt B., Keller M., Oelschlegel A., McIntyre L., Groschup M.H. (2012). Spread of classic BSE prions from the gut via the peripheral nervous system to the brain. Am. J. Pathol..

[38] Wells G.A., Spiropoulos J., Hawkins S.A., Ryder S.J. (2005). Pathogenesis of experimental bovine spongiform encephalopathy: Preclinical infectivity in tonsil and observations on the distribution of lingual tonsil in slaughtered cattle. Vet. Rec..

[39] Baron T., Vulin J., Biacabe A.G., Lakhdar L., Verchere J., Torres J.M., Bencsik A. (2011). Emergence of classical BSE strain properties during serial passages of H-BSE in wild-type mice. PLoS One.

[40] Beringue V., Andreoletti O., Le Dur A., Essalmani R., Vilotte J.L., Lacroux C., Reine F., Herzog L., Biacabe A.G., Baron T., Caramelli M., Casalone C., Laude H. (2007). A bovine prion acquires an epidemic bovine spongiform encephalopathy strain-like phenotype on interspecies transmission. J. Neurosci..

[41] Capobianco R., Casalone C., Suardi S., Mangieri M., Miccolo C., Limido L., Catania M., Rossi G., Di Fede G., Giaccone G., Bruzzone M.G., Minati L., Corona C., Acutis P., Gelmetti D. (2007). Conversion of the BASE prion strain into the BSE strain: The origin of BSE?. PLoS Pathog..

[42] Benestad S.L., Mitchell G., Simmons M., Ytrehus B., Vikoren T. (2016). First case of chronic wasting disease in Europe in a Norwegian free-ranging reindeer. Vet. Res..

[43] Babelhadj B., Di Bari M.A., Pirisinu L., Chiappini B., Gaouar S.B.S., Riccardi G., Marcon S., Agrimi U., Nonno R., Vaccari G. (2018). Prion disease in Dromedary camels, Algeria. Emerg. Infect. Dis..

[44] Bischof J., Maeda R.K., Hediger M., Karch F., Basler K. (2007). An optimized transgenesis system for Drosophila using germ-line-specific phiC31 integrases. Proc. Natl. Acad. Sci. U. S. A..

[45] Green C., Levashina E., McKimmie C., Dafforn T., Reichhart J.M., Gubb D. (2000). The necrotic gene in Drosophila corresponds to one of a cluster of three serpin transcripts mapping at 43A1.2. Genetics.

[46] Lacroux C., Vilette D., Fernandez-Borges N., Litaise C., Lugan S., Morel N., Corbiere F., Simon S., Simmons H., Costes P., Weisbecker J.L., Lantier I., Lantier F., Schelcher F., Grassi J. (2012). Prionemia and leukocyte-platelet-associated infectivity in sheep transmissible spongiform encephalopathy models. J. Virol..

[47] Kupfer L., Eiden M., Buschmann A., Groschup M.H. (2007). Amino acid sequence and prion strain specific effects on the *in vitro* and *in vivo* convertibility of ovine/murine and bovine/murine prion protein chimeras. Biochim. Biophys. Acta.

[48] Feraudet C., Morel N., Simon S., Volland H., Frobert Y., Creminon C., Vilette D., Lehmann S., Grassi J. (2005). Screening of 145 anti-PrP monoclonal antibodies for their capacity to inhibit PrPSc replication in infected cells. J. Biol. Chem..

[49] White K.E., Humphrey D.M., Hirth F. (2010). The dopaminergic system in the aging brain of Drosophila. Front. Neurosci..

[50] Beck K.E., Chaplin M., Stack M., Sallis R.E., Simonini S., Lockey R., Spiropoulos J. (2010). Lesion profiling at primary isolation in RIII mice is insufficient in distinguishing BSE from classical scrapie. Brain Pathol..

