# Supplementary material for: A new model for sensitive detection of zoonotic prions by PrP transgenic Drosophila
Source: J Biol Chem. 2021 Jul 13;297(2):100878. doi: 10.1016/j.jbc.2021.100878 (PMC8350378; doi:10.1016/j.jbc.2021.100878)

Legends for Supporting information

Figure S1. Serial transmission of ovine BSE prion seeding activity in bovine PrP *Drosophila*
Ovine passaged BSE or control prion-free bovine material was passaged in *Elav* x bovine PrP or *Elav* x 51D (51D) *Drosophila* (primary passage) and subsequently in *Elav* x bovine PrP *Drosophila* (secondary passage). At various times after hatching, head homogenate was prepared from harvested secondary passage bovine PrP *Drosophila* and used as seed in PMCA reactions. Western blot was used to detect PK-resistant PrP27-30 in PMCA reaction products seeded with ovine-BSE-exposed *Drosophila* head homogenate. Molecular mass markers in kDa shown on the left. WB control = western blot control comprising PG127 scrapie-infected sheep brain material included to highlight the low molecular weight band of un-glycosylated BSE PK-resistant PrP^{Sc}. C-BSE = classical BSE.

Figure S2. Prion seeding activity in classical BSE-exposed bovine PrP *Drosophila*
Elav x bovine PrP and *Elav* x 51D *Drosophila* were exposed at the larval stage to 10^{-2} - 10^{-14} dilutions classical BSE-infected or prion-free control bovine brain material. At various times after hatching, head homogenate was prepared from harvested flies and used as seed in PMCA reactions. Prion seeding activity in PMCA reaction products seeded with head homogenate prepared from classical BSE-exposed or control *Drosophila* was analysed by western blot for detection of PK-resistant PrP27-30. Molecular mass markers in kDa shown on the left. WB control = western blot control comprising PG127 scrapie-infected sheep brain material included to highlight the low molecular weight band of un-glycosylated BSE PK-resistant PrP^{Sc}.

Figure S3. Accelerated loss of locomotor ability in classical BSE-exposed bovine PrP *Drosophila*
Adult Elav x bovine PrP (Figure S3a) and Elav x 51D (Figure S3b) *Drosophila* were assessed for their locomotor ability by a negative geotaxis climbing assay following exposure at the larval stage to 10^{-2} - 10^{-14} dilutions of classical BSE-infected bovine brain homogenate. Control inoculum was a 10^{-2} dilution of prion-free bovine brain homogenate (Control 10^{-2}). The data shown are linear regression plots of the mean performance index ± SD for three groups of flies per time point calculated as described in the Experimental Procedures. C-BSE = classical BSE.

Table S1. Reduced median survival time in classical BSE-exposed bovine PrP *Drosophila*
Adult *Elav* x bovine PrP *Drosophila* were assessed for their survival following exposure at the larval stage to 10^{-2} - 10^{-20} dilutions of classical BSE-infected bovine brain homogenate. Control inoculum was a 10^{-2} dilution of prion-free bovine brain homogenate. The data shown are median survival time in days with statistical analysis carried out using Kaplan-Meier plots. * *p* value for response with Control 10^{-2} compared to response of each dilution of classical BSE.

Figure S4. Locomotor response of 51D non-transgenic control *Drosophila* after exposure to H- or L-type atypical BSE or ovine BSE
Adult *Elav* x 51D *Drosophila* were assessed for their locomotor ability by a negative geotaxis climbing assay following exposure at the larval stage to dilutions (a) H-type BSE; (b) L-type BSE; or (c) ovine BSE-infected bovine brain homogenate. Control inoculum was a 10^{-2} dilution of prion-free bovine brain homogenate (Control 10^{-2}). The data shown are linear regression plots of the mean performance index ± SD for three groups of flies per time point calculated as described in the Experimental Procedures.

Figure S5. Detection of disease-associated PrP by immunohistochemistry in the brain of classical BSE challenged tg110 mice

A characteristic feature of classical BSE-infected tg110 mice is the formation in the brain of plaques of disease-associated PrP that tend to form linear arrangements. These plaques were observed in tg110 mice challenged with (a) 10^{-1}; (b) 10^{-2} and (c) 10^{-4} dilutions of classical BSE inoculum but absent in (d) unchallenged control mice. (a); (b) and (d) midbrain; (c) medulla. Scale bar represents 50µm.

Figure S1. Serial transmission of ovine BSE prion seeding activity in bovine PrP *Drosophila*

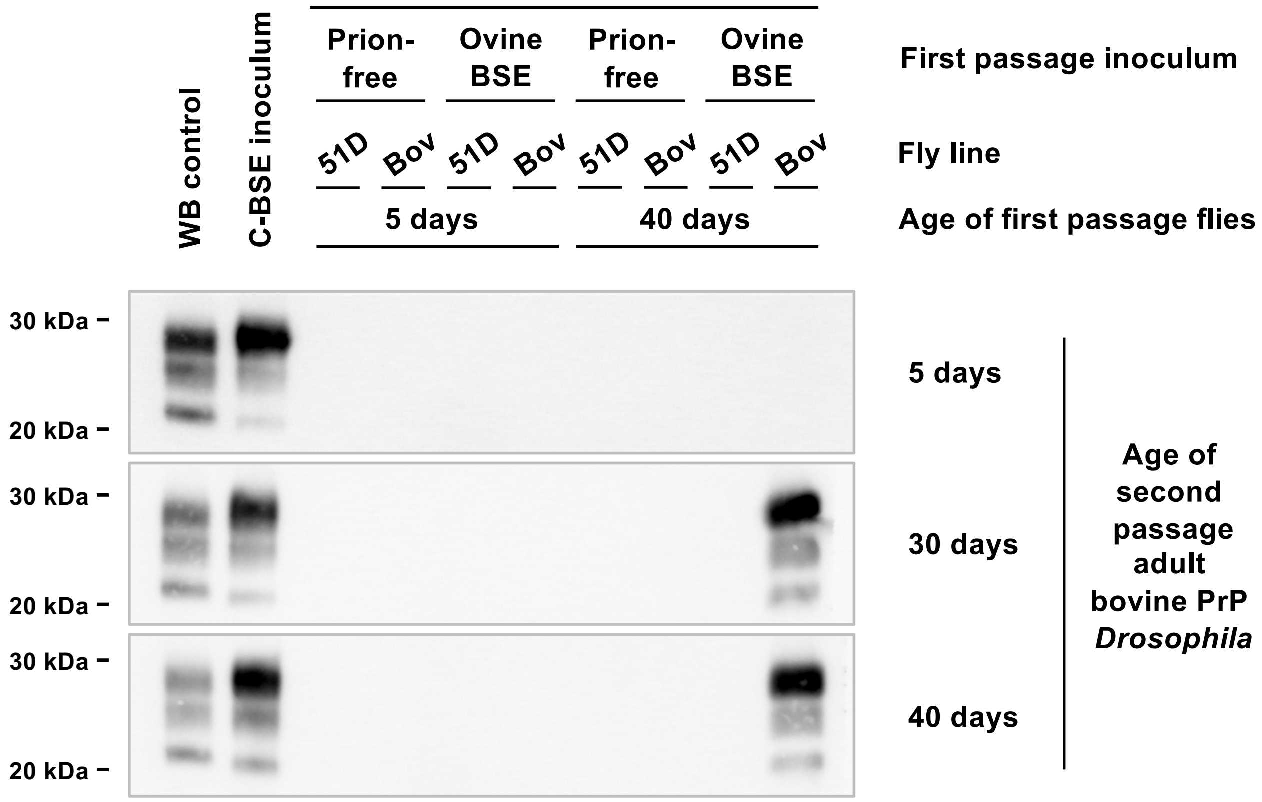

Figure S2. Prion seeding activity in classical BSE-exposed bovine PrP *Drosophila*

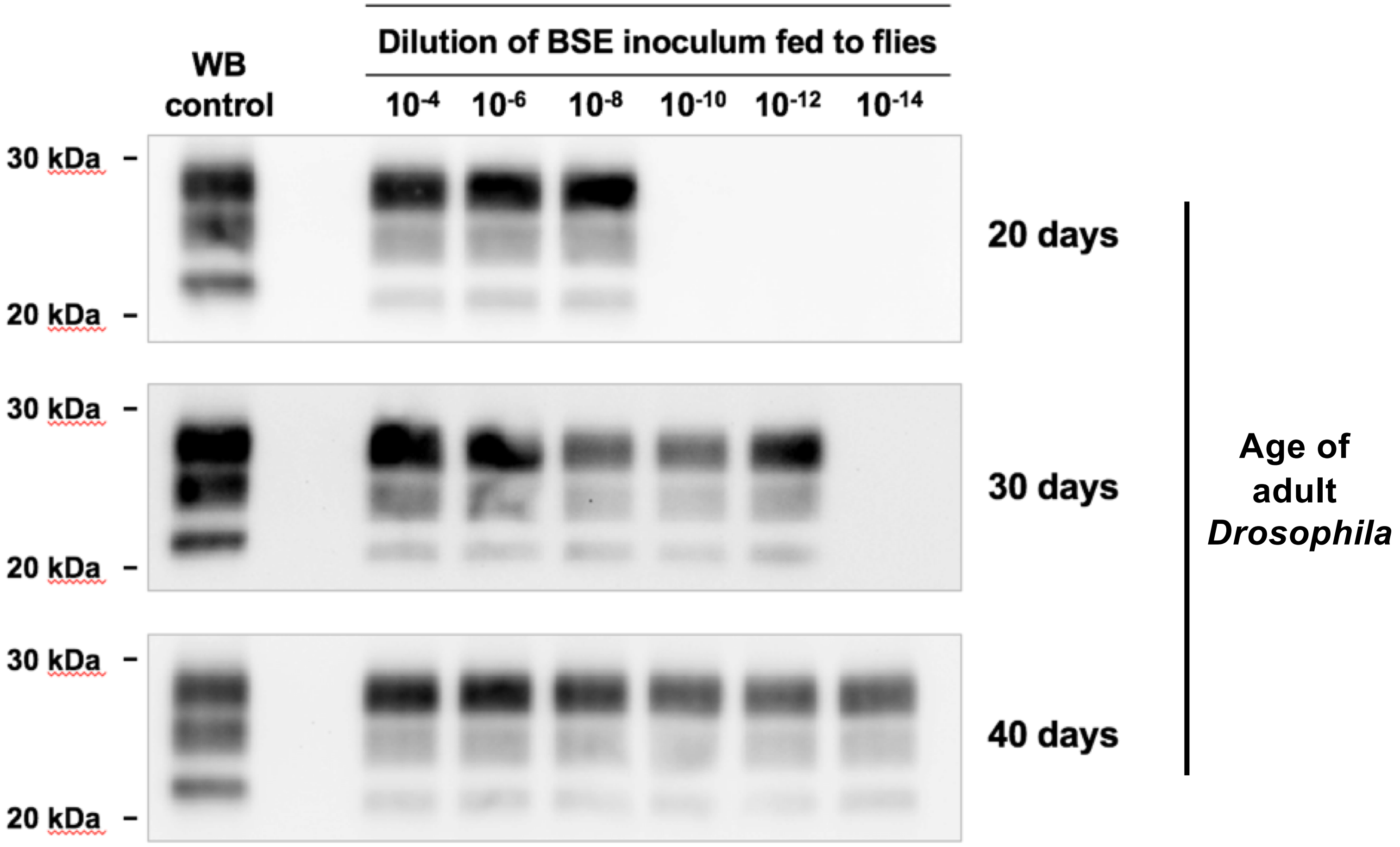

Figure S3. Accelerated loss of locomotor ability in classical BSE-exposed bovine PrP *Drosophila*

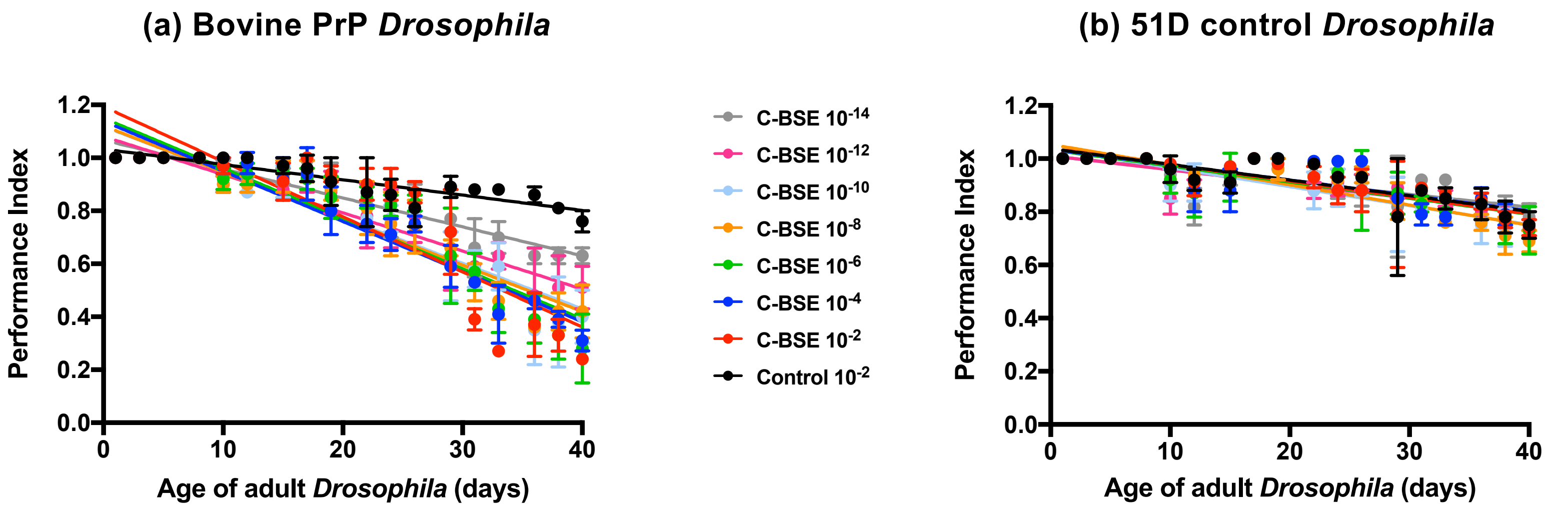

Table S1. Reduced median survival time in classical BSE-exposed bovine PrP *Drosophila*

	Control	Dilution of classical BSE inoculum (log_{10})									
	10^{-2}	10^{-2}	10^{-4}	10^{-6}	10^{-8}	10^{-10}	10^{-12}	10^{-14}	10^{-16}	10^{-18}	10^{-20}
Median survival (days)	127	59	66	75	82	94	103	122	129	129	127
p value*		<0.0001	<0.0001	<0.0001	<0.0001	<0.0001	<0.0001	0.0027	>0.05	>0.05	>0.05

Figure S4. Locomotor response of 51D non-transgenic control *Drosophila* after exposure to H- or L- type atypical BSE or ovine BSE

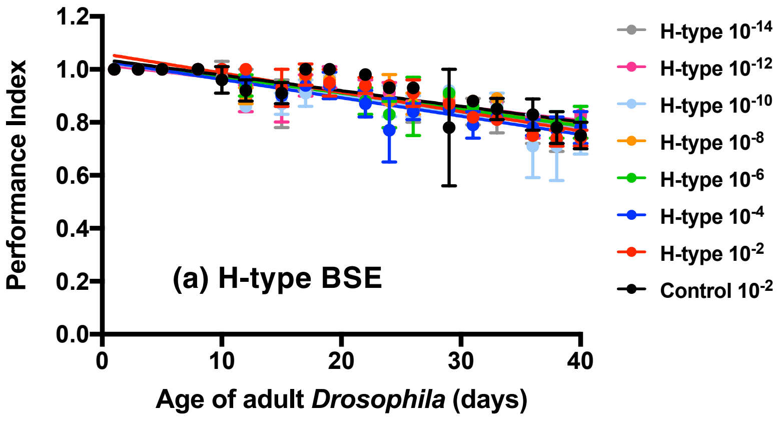

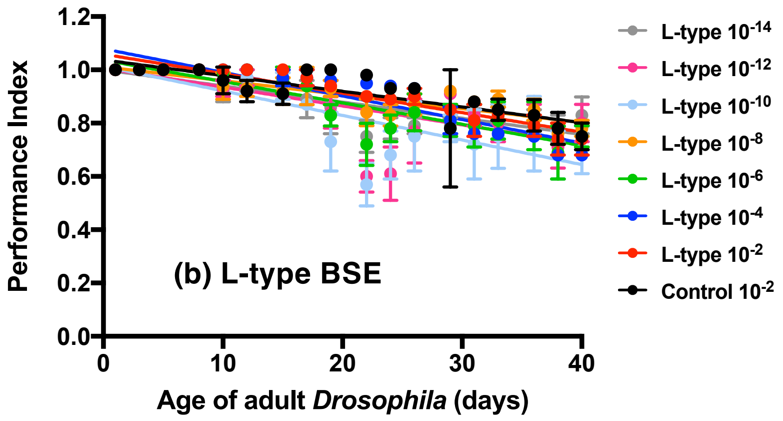

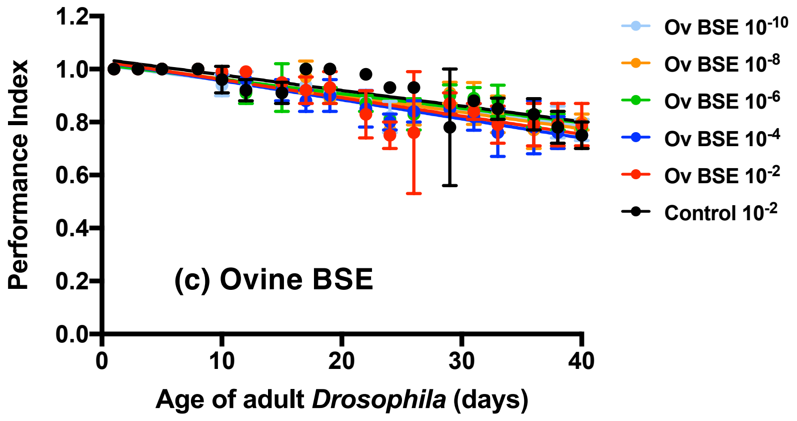

Figure S5. Detection of disease-associated PrP by immunohistochemistry in the brain of classical BSE challenged tg110 mice

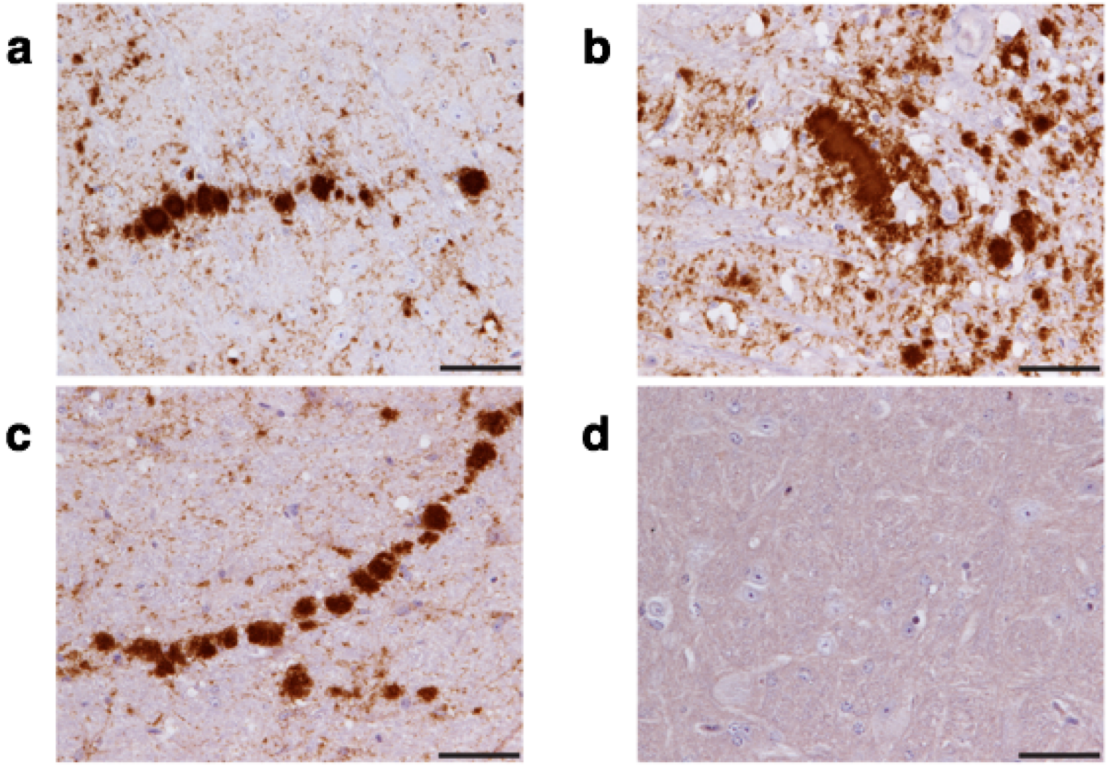

Supplement: Supplemental Figures S1–S5 and Table S1 [file mmc1.pdf]
